# Distinctive properties of biological neural networks and recent advances in bottom-up approaches toward a better biologically plausible neural network

**DOI:** 10.3389/fncom.2023.1092185

**Published:** 2023-06-28

**Authors:** Ikhwan Jeon, Taegon Kim

**Affiliations:** Brain Science Institute, Korea Institute of Science and Technology, Seoul, Republic of Korea

**Keywords:** bottom-up approach, biologically plausible neural network, optimization of neural network, biological neural network supremacy, neural network architecture, balanced network, dendritic computation, Dale's principle

## Abstract

Although it may appear infeasible and impractical, building artificial intelligence (AI) using a bottom-up approach based on the understanding of neuroscience is straightforward. The lack of a generalized governing principle for biological neural networks (BNNs) forces us to address this problem by converting piecemeal information on the diverse features of neurons, synapses, and neural circuits into AI. In this review, we described recent attempts to build a biologically plausible neural network by following neuroscientifically similar strategies of neural network optimization or by implanting the outcome of the optimization, such as the properties of single computational units and the characteristics of the network architecture. In addition, we proposed a formalism of the relationship between the set of objectives that neural networks attempt to achieve, and neural network classes categorized by how closely their architectural features resemble those of BNN. This formalism is expected to define the potential roles of top-down and bottom-up approaches for building a biologically plausible neural network and offer a map helping the navigation of the gap between neuroscience and AI engineering.

## 1. Introduction

Turing's idea of building a thinking machine by replacing an organism with artifacts, part by part (Turing, 1948), has inspired scientists and engineers because it was the first clear statement of a bottom-up approach toward building artificial intelligence (AI). In general, the term “bottom-up” refers to the directionality of an approach that begins with specifics or minutiae to arrive at a comprehensive solution. Thus, the bottom-up approach to developing a brain-like intelligence system begins with spatiotemporal local properties and their organized combinations. Local properties are presented in neurons or synapses, namely, single computational units and their combinations directly depict the connectivity and architecture of a neural circuit. Because these details and their effects are covered by the discipline of neuroscience, developing AI from the ground up using an understanding of neuroscience is straightforward. However, even the latest neuroscience field lacks comprehensive knowledge of neural circuits, their functions, and the mapping between them, indicating that the operating principle of neural networks is absent in practice (Goodfellow et al., 2016; Jonas and Kording, 2017). Thus, experimental attempts to translate up-to-date piecemeal information on various characteristics of neurons, synapses, and neural circuits into AI are the only viable options under these circumstances. Given that replacing a component of an artificial neural network (ANN) with the counterpart of a biological neural network (BNN) generally does not outperform the original ANN and is often not very influential, a bottom-up approach appears to be infeasible and impractical although it does not imply inherent impossibility, as Turing contested (Turing, 1948).

Nonetheless, we believe exploring the gap between neuroscience and AI engineering using a bottom-up approach should be encouraged. Although no unified principle governing multiscale neural network features has been found, there are several useful models describing phenomena at different scales. Good examples include the Hebbian learning principle and its modifications, encompassing various forms of long-term synaptic plasticity (Dayan and Abbott, 2001). Considering the history of AI development, it is unsurprising that an ANN incorporates specific principles from neuroscience and computational neuroscience. The birth of successful modern approaches, such as deep neural networks and their learning algorithms, is partly attributable to this type of strategy (Goodfellow et al., 2016). Furthermore, given the massive amount of resources required to operate such systems (Schuman et al., 2022), further information behind the efficient computation by the BNN should be uncovered and implanted into the ANN. To accelerate exploration using a bottom-up approach, cooperation between neuroscientists and AI engineers can be promoted through mutual benefits. One of the goals of neuroscience is to reveal the neural network mechanisms underlying a particular mental state or behavior that the neural network principle can encapsulate. This process requires confirmation by observations made in a controlled setting or laboratory experiments; however, because of their complexity, the brain and neural circuits are often inaccessible in a properly controlled manner. Furthermore, confirming a unified operating mechanism is challenging because of the low practicality of long-term and large-scale manipulation of the brain and neural system. AI engineering can serve as a valuable analogical model spanning several spatiotemporal scales, from a cellular level to behavioral consequences. Hence, an ANN based on the BNN features provides a proof-of-concept for a particular neural network principle, demonstrating how a neural circuit produces a specific behavior. On the other hand, the neural network principle contributes to a better understanding of how ANNs work. Considering that currently successful ANNs require improved explainability and interpretability (Gunning et al., 2019; Vilone and Longo, 2021; Nussberger et al., 2022), bottom-up approaches equipped with neural network principles can help AI designers better understand the outcomes of their ANN models. Thus, this review preferentially introduces studies that focused on the conceptual similarity between the components of a given ANN and its corresponding BNN, regardless of the model's performance on the tasks designed for ANNs.

On the other hand, because other types of approaches toward well-functioning intelligence systems have been successful, such as the recent advancement of large-scale language models (Devlin et al., 2019; Brown et al., 2020) and text-to-image models (Ramesh et al., 2022; Rombach et al., 2022), approaches under purely engineering goals seem to dispense with the need for a bottom-up approach. However, such approaches merely offer an explanation of how the brain is capable of many cognitive functions with BNN, contrary to the mutual benefit expected from the bottom-up approach. Top-down approaches like “brain-inspired” AI (Chen et al., 2019; Robertazzi et al., 2022; Zeng et al., 2022) partly enhance our understanding of the brain, especially the cognitive process of a certain task, and improve performances simultaneously, whereas their goals do not reach the circuit-level mechanism of BNN. At the other extreme, attempts to emulate BNN have been made to copy a mesoscopic neural circuit and demonstrate that copied BNN indeed show the same activity measured from experiments (Markram et al., 2015). They are useful for replacing invasive experiments in the future and for simulating virtually controlled experiments. However, these detailed models are not directly applicable to AI systems because of their low cost-effectiveness and relatively simple output pattern, despite large-scale computation with a large number of parameters to be optimized. Therefore, this review focuses on studies that consider the mutual benefits between scientific and engineering goals at the proper level of BNN abstraction.

**Figure 1:**
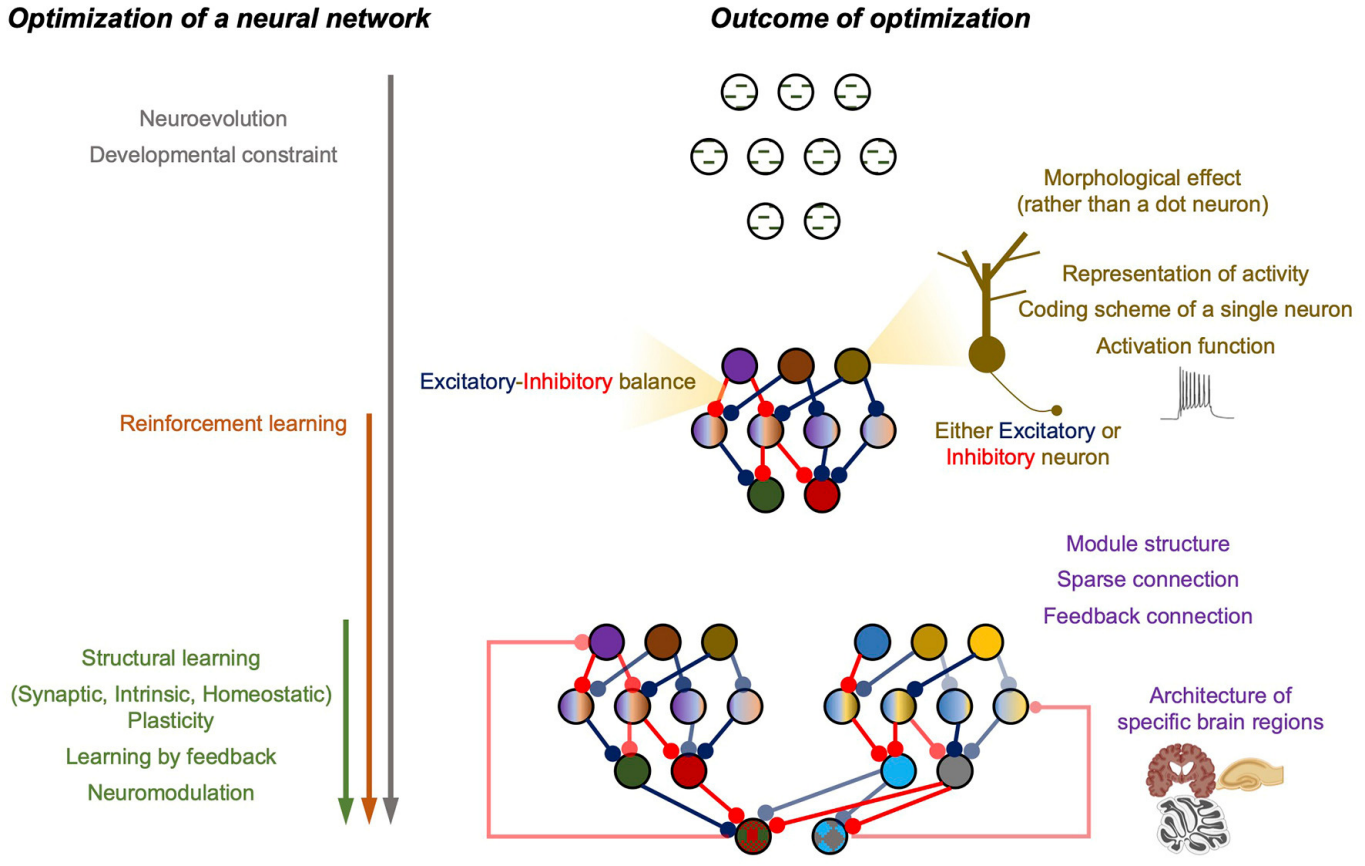
Summary figure of the review. **(Left)** The optimization processes of a neural network. Arrows represent the involvement of each process with time. **(Right)** The outcome of the optimization.

Considering the rudiments of deep neural networks, the first step was to construct a neural network and select a training algorithm after determining the task and training dataset. Unlike ANN, nature handles the search for a BNN architecture and builds a training strategy. Thus, we begin the review with ANN's architecture search and training algorithm, which was inspired by the natural process of network structure optimization and its updates. As the optimization process continues, the properties of the single computational units and the architecture of the neural circuit are updated, which can be viewed as the outcome of successful optimization. This implies that understanding BNN properties and their impact on computation can be advantageous because such properties of BNN studied have already been refined by nature. Hence, the following sections of this review focus on the montage of useful BNN properties and the efforts related to the direct utilization of BNN properties in ANN design (summarized in Figure 1).

To develop a systematic manner as opposed to a random search of proper links between neuroscience and AI engineering, we defined the set of objectives that neural networks try to achieve as “the problem space” and categorized neural network models based on how closely their architectural features resemble those of BNN. Such formalization may offer an approximate map, including the limitations of ANN and what we should aim for when constructing a biologically plausible neural network. Using this map, we proposed the potential roles of neuroscience and AI engineering and their cooperative workflow pipeline. We believe that this pipeline will encourage reciprocal advantages by demonstrating how top-down and bottom-up approaches from neuroscience can offer useful information for AI engineering and, conversely, how AI engineering advances our understanding of the brain and its function.

## 2. Optimization strategy: multiscale credit assignment

All biologically intelligent agents interact with their environments and attempt to survive and reproduce. A combination of hereditary mutations and epigenetic adaptations builds up a biological agent's fitness, and the agents are eventually evaluated for survival (or death) and reproduction (or nonproliferation). One of the essential organs in an individual agent is the brain, which is optimized using the same process (Tosches, 2017). Although the entire optimization process can be understood in parts by dividing it into different temporal scales, each part still encounters the common conundrum of how much each spatiotemporal local parameter should be updated to improve fitness. Thus, this issue can be described as a multiscale credit assignment problem (Valiant, 2013). Assuming that the properties of the computational units, network architecture, and overall performance of the network are the outcomes of BNN optimization, it is worthwhile to imitate this strategy to achieve superior biologically plausible neural networks. In this review, we simply hypothesized that a longer time-scale optimization relates to the architectural search process through evolution and development, whereas a shorter-scale optimization corresponds to the learning process in a neural circuit or brain.

### 2.1. Architecture search: evolution and development

The process of evolution includes the development and learning of a neural circuit; therefore, it is a credit assignment process with the longest temporal scale. Genes that must be evaluated for fitness are prepared by mutations, and the neural circuit variants built from these genes are eventually tested by natural selection (Tosches, 2017; Hasson et al., 2020). The artificial counterpart of the mutation-selection process, namely, the evolutionary algorithm (EA), has been applied in numerous domains for decades, and “neuroevolution” refers to the application of EA to neural networks (Yao and Liu, 1998; Stanley et al., 2019; Galván and Mooney, 2021). Although the neuroevolution scheme simplified or omitted numerous aspects of the biological evolution process, it successfully captured the essentials and performed well in rediscovering the BNN properties (Risi and Stanley, 2014) and optimizing the ANN architecture (Liang et al., 2018; Zoph et al., 2018). In addition to structural connectivity, network architecture comprises the functional features of a network, such as the activation function of each neuron and its hyperparameters or initial synaptic weights. For example, the hyperparameters of different neuronal activation functions can be optimized using the EA (Cui et al., 2019). In deep learning, EA and reinforcement learning have been widely employed for the automated network model selection, termed neural architecture search (NAS; Elsken et al., 2019; Liu Y. et al., 2021).

In a BNN, developmental processes add diversity or constraints to neural networks through their stochastic nature or spatial arrangement, respectively (Smith, 1999; Tosches, 2017; Luo, 2021), in addition to a genetic code-driven architecture search. During development, neurons are ready to grow and connect to others, controlled by internally produced proteins (genetic codes) and external cues. Biological studies have revealed sequentially proceeding developmental processes: neuralation, proliferation, cell migration, differentiation, synaptogenesis, synapse pruning, and myelination (Tierney and Nelson, 2009). The first three steps indicate the orchestrated positioning of neuronal nodes in space, and the consecutive processes drive the formation of proper connections. Although genetic codes can drive the overall coordination of neuronal nodes in a three-dimensional space, chemical cues, such as morphogens, are constantly exposed to stochastic fluctuations (van Ooyen, 2011; Goodhill, 2018; Razetti et al., 2018; Llorca et al., 2019; Staii, 2022). Additionally, considering that synaptogenesis induces the randomly generated overproduction of synapses and connectivity is polished by pruning and myelination processes (van Ooyen, 2011; Goodhill, 2018; Razetti et al., 2018), the potential intervention of probabilistic diversification to differentiate connectivity is highly likely. Such stochasticity depends on the environment to which the brain is exposed. Thus, the common skeleton of the BNN architecture across individuals is an essential structure of a neural network to perform naturalistic tasks stably, and the variability in each individual agent is a sign of adaptation to different environments. This implies that by introducing such variability, we may be able to expand the range of searches in the parametric space of a neural network compared with relying only on genetic codes and mutations.

Although the evolution and development of BNN have potential advantages during ANN construction, direct and thorough imitation of these processes does not necessarily guarantee better ANN performance. First, when nature searches for answers through evolution and development, it utilizes an extremely efficient parallel search by preparing variable groups of individuals and combinations between groups (Foster and Baker, 2004; Traulsen and Nowak, 2006). To emulate such a process on a conventional computer, each individual needs to be stored in memory and evolved through a series of calculations, greatly increasing the computational burden. Thus, some processes should be simplified, and we need to capture the essential parts like the neuroevolution approach although an ensemble neural network strategy that shares the concept of group selection has been applied to construct and optimize ANN (Krogh and Vedelsby, 1994; Zhou et al., 2002; Liu and Yao, 2008; Zhang S. et al., 2020). The second aspect is platform dependency; as mentioned above, the optimization processes occurring in the brain depend on the spatial arrangement of computing units and chemicals as well as genetic codes, which implies that the distance between neurons can limit wiring (van Ooyen, 2011; Goodhill, 2018). Because the spatial arrangement of neurons and wiring costs do not matter in the simulation of an ANN, the direct translation of evolution and developmental processes for the BNN is not an effective option. Thus, only when we construct an ANN on a platform where the wiring cost can be defined, the emulation of the BNN formation through the direct imitation of evolution and development may offer a better architecture search algorithm. Third, evolution and development are primarily driven by the environment. In contrast to the well-specified task and dataset in ANN, the environment to which the BNN has to adapt is vast and carries an intensive amount of information, which blurs the boundary of essential information for training specific neural circuits. A notable recent study circumvented these problems and demonstrated that simplified developmental and evolutionary processes can select a biologically plausible neural circuit (Hiratani and Latham, 2022). This study utilized a rather simple feedforward neural network to approximate olfactory information in an environment, which was considered a teacher network to train a student network that corresponds to a biological olfactory circuit consisting of expansion-contraction coding architecture; eventually, such a simple approach successfully met with the scaling laws in BNN. This study showed a model case of how a mutually beneficial investigation can be designed to enhance the understanding of both BNN and ANN.

### 2.2. Learning algorithm

Once the fundamental architecture is determined by genetic codes and developmental processes, as described above, the BNN begins to be rapidly trained by interacting with the environment. Both structural and functional changes are involved in the biological implementation of this training process, which we call learning. Structural changes include neurogenesis, neuronal death, synaptogenesis, and pruning, while functional changes indicate the plasticity of neurons and synapses in the brain. Considering that local chemical and physiological mechanisms mediate these changes, achieving global adaptation through learning is a problem that the BNN must resolve, which we refer to as the populational credit assignment problem of computing units (Friedrich et al., 2011; Zou et al., 2023). Additionally, when instruction information for a proper change is provided by a circuit mechanism, such as a feedback connection, it is accompanied by an unavoidable delay that eventually causes a temporal credit assignment problem (Friedrich et al., 2011; Zou et al., 2023).

#### 2.2.1. Local attributes: structural changes

Structural changes in the brain occur throughout the lifespan of an animal. However, considering that neurogenesis is a rare event and is observed in confined brain regions in adults, if any (Sorrells et al., 2018, 2021; Abdissa et al., 2020; Moreno-Jiménez et al., 2021), and significant neuronal death is expected to take place in old age or a pathological brain (Mattson and Magnus, 2006), simply assuming that the number of nodes of a neural network is determined by development is in the range of biological plausibility. In brain regions where we can expect significantly observable neurogenesis, such as the dentate gyrus in the hippocampus, a notable study reported that newly added neuronal nodes could contribute to neural network performance by working as a neural regularizer to avoid overfitting (Tran et al., 2022). In contrast to the addition of neuronal nodes, neuronal death may be superficially interpreted as the negative regulation of neural networks as observed in the aging or degenerative pathology of the brain (Mattson and Magnus, 2006). However, considering that some cognitive features can improve with age (Murman, 2015; Veríssimo et al., 2022), well-regulated neuronal death may not directly indicate the total dysfunction of a neural network. Two potential biological mechanisms account for this paradoxical positive regulation by removing neuronal nodes. First, as observed in biological studies (Kuhn et al., 2001; Merlo et al., 2019) and implied by computational studies (Barrett et al., 2016; Tan et al., 2020; Terziyan and Kaikova, 2022), a biological system often prepares compensatory mechanisms against sudden changes that can function as a temporary or partial advantage in neural computation. Another possibility is the intrinsic advantage achieved by removing neuronal nodes. In ANN, similar negative structural regulations have already been utilized as a form of “drop-out” or “sparsification” by intentionally removing neuronal nodes (Goodfellow et al., 2016; Tan et al., 2020; Hoefler et al., 2022). Because cognitive advantage with gradually increased neuronal death and its circuit mechanisms are largely unexplored, ANNs that include neuronal deaths and show partially or temporarily improved performances can offer new insights for both neuroscience and AI engineering.

Unlike the structural changes caused by neuronal addition or removal, new synapse formation and synaptic elimination by pruning, which are the addition and removal of edges in a neural network, occur more generally in the brain. The axon of a presynaptic neuron and the dendrite of a postsynaptic neuron should be within a proper distance before making a new synapse, and then a new synapse can be formed by the Hebbian type activity-dependent synaptogenesis (Südhof, 2018). However, the local mechanism of edge addition is insufficient for the optimization of an entire network and can result in excessive connectivity redundancy between activity-correlated neurons unless there is a regulatory mechanism. To participate in the optimization of a neural network, neurons must utilize information other than local synaptic activity. Negative regulatory mechanisms, such as synaptic elimination, are required to properly adjust the number of edges, which is widely utilized as an algorithm for the sparsification of a neural network to reduce the model (Luo, 2020; Hoefler et al., 2022). Adaptive synaptogenesis (Miller, 1998; Thomas et al., 2015), reinforcement signals from reward and punishment (Dos Santos et al., 2017), or other types of neuromodulation (Garcia et al., 2014; Speranza et al., 2017) may achieve such orchestration between positive and negative regulation. The counterparts of edge number regulation by synapse formation and elimination in ANN are the additive update of a synaptic weight from zero-weight connection and making a synaptic weight to zero, respectively, implying that the structural changes in synapses can be interpreted as the on-off switch type of functional changes. Interestingly, beyond the dichotomy of synapses or no synapses, a contact point between two neurons is ready to be switched on by the Hebbian-type learning rule in the form of a silent synapse (Kerchner and Nicoll, 2008; Hanse et al., 2013), which is also found in filopodia lacking AMPA receptors and containing NMDA receptors in the adult neocortex (Vardalaki et al., 2022). Considering that the brain should adapt to an increase in the amount of information to be stored, such a substrate for readiness is a valuable mechanism (Fusi et al., 2005; Vardalaki et al., 2022). Additionally, because a stable consolidation of acquired information into already stored information is accompanied by the rearrangement of the synaptic weights and connectivity, on- and off-type regulation should be appropriately utilized (Jedlicka et al., 2022). For ANN simulation on the current computer form, zero-weight synapse costs roughly the same as any other weight value in the allowed range; however, in BNN, physical wiring and its maintenance require additional resources. Hence, when constructing an ANN on a platform where the cost can be reduced by eliminating connections, a NAS strategy must be considered based on various types of structural changes in the BNN.

Similar to our categorization, a recent review (Maile et al., 2022) also regarded these structural changes after the developmental period as “structural learning,” which implies that NAS across a multi-temporal scale needs to continue for the whole life. In summary, structural changes in a neural network achieved by controlling the number of neurons or synapses are the key concepts that optimize a neural network architecture during its lifespan, and their implementation in an ANN can contribute to the construction of a better-performing neural network with reduced resource requirements under specific platforms.

#### 2.2.2. Local attributes: functional changes

Although functional changes in a neural network are less explicit than physically expressed structural changes, they occur much more often in the brain and are essential for the fineness of adaptation. Various types of plasticity occurring at synapses or neurons are key components of the functional changes in a neural network.

Considering that a neuron transmits information as a spiking electrical signal, the so-called action potential, any change that alters the probability of generating action potentials under the same input indicates a change in neuronal excitability, which is called intrinsic plasticity. Thus, the intrinsic plasticity of a neuron can be interpreted as a transition from a certain state of neuronal excitability to a different state (Titley et al., 2017; Debanne et al., 2019). In a BNN, the concept of intrinsic plasticity is suitable for implementing memory mechanisms. Input-dependent stable changes in neuronal excitability can be directly paired with the hypothesis of the cellular level memory engram (Titley et al., 2017; Alejandre-García et al., 2022). Additionally, because the parameters of synaptic plasticity are significantly affected by the average activities of both pre- and post-synaptic neurons, as indicated by the Bienenstock-Cooper-Munro (BCM) model (Bienenstock et al., 1982; Dayan and Abbott, 2001), intrinsic plasticity can also be interpreted as a means of metaplasticity (Sehgal et al., 2013). Thus, implementing intrinsic plasticity in an ANN can improve the representability of the given information. In an ANN, the concept of neuronal excitability is expressed as a bias before the activation function determines the neuron's output. In many ANN cases, bias is considered a common constant within a layer or even set to zero. We can expect significantly better performances by introducing intrinsic plasticity into ANN or spiking neurons (Zhang and Li, 2019; Zhang et al., 2019). A similarity between the simplified intrinsic plasticity introduced in ANN and batch normalization has also been reported (Shaw et al., 2020).

The concept of synaptic plasticity involves changes in the efficacy of synaptic transmission across multiple temporal scales. Because a neuron propagates information through spikes, the main mechanism of synaptic plasticity is expected to depend on spike timing rather than amplitude by considering a uniform voltage level of action potential firing. Although the extent to which the synaptic weight should be adjusted depending on the timing differences between the presynaptic spike and postsynaptic spike varies with neuronal types, synaptic properties, or the existence of neuromodulation, synaptic plasticity occurring timing difference can be categorized as spike-timing-dependent-plasticity (STDP; qiang Bi and ming Poo, 1998). Under an ultra-sparse firing regime, STDP may be the sole mechanism to implement synaptic plasticity, which features Hebbian plasticity, in which neurons that fire together wire together (Song et al., 2000; Caporale and Dan, 2008). However, because information encoding is not always at the level of a single action potential firing, the description of synaptic plasticity at the level of each spike cannot explain the computational implications of the consequences of such plasticity. Thus, it is necessary to build a description of synaptic plasticity that depends on momentary information transmitted through the synapse. Rate-dependent encoding occurs at a longer timescale or under a denser spiking regime (Gerstner et al., 1997). Classical computational neuroscience has already depicted such plasticity by formalizing and improving Hebbian plasticity using additional terms (Dayan and Abbott, 2001). In fact, Hebbian plasticity and its variants could well describe synaptic plasticities in BNN, and by introducing the concept of sliding threshold, metaplasticity could be incorporated into formalism (Abraham, 2008; Laborieux et al., 2021). However, because these phenomenological models focus on simple but accurate descriptions of various synaptic plasticities in BNN, they require ad hoc terms or modifications if more diverse dynamics in synaptic plasticity and metaplasticity are observed. In contrast, mechanistic models can be more useful for generalizing various types of synaptic plasticity by introducing the dynamics of biological synaptic components. For example, considering that short-term plasticity can be utilized to stably represent information for a certain short period in a buffer-like neural network, analogous cognitive mechanisms such as working memory can be modeled (Masse et al., 2019), which may open up more promising future applications to the artificial memory system by introducing more detailed synaptic components. Indeed, a mechanistic model for short-term plasticities, such as the Tsodyks-Markram model (Tsodyks and Markram, 1997), could be utilized to explain working memory modulation (Rodriguez et al., 2022) and may help to build a better neuromorphic device (Zhang et al., 2017; Li et al., 2023) or a better artificial working memory system (Averbeck, 2022; Kozachkov et al., 2022; Rodriguez et al., 2022). The mechanical description of long-term synaptic plasticity is often composed of several processes responsible for multiple-timescale mechanisms, as indicated in the cascade model of binary switches constructed using positive feedback loops with multiple time constants (Kawato et al., 2011; Helfer and Shultz, 2018; Smolen et al., 2020). Although the readout of biological synaptic plasticity is the same as the weight adjustment in ANN, such mechanistic models may largely help construct a new type of metaplasticity algorithm in ANN. Considering the recent spotlight on metaplasticity as one of the solutions to catastrophic forgetting (Jedlicka et al., 2022), it has become more important to understand how synapses in BNN can form their metastable states and how synaptic plasticity can exploit the transition between these states to enhance the representation of information (Fusi et al., 2005; Benna and Fusi, 2016; Abraham et al., 2019).

#### 2.2.3. Global optimization

An orchestrated strategy is required for these local processes of plasticity to result in the learning of a certain function. Learning is the adaptation of a neural network to approximate a function that maps from the input from the environment to the target output, which is a global optimization process (Zhang H. et al., 2020). The optimization target and the algorithm for efficiently reaching the target by combining local processes should be elucidated to define this optimization. Although how the brain can optimize neural networks and what kind of target it tries to minimize or maximize are generally unknown, there are several phenomena observed in BNN that can be the hint or the starting point toward building biologically plausible optimization algorithms. For example, homeostatic control of neuronal activity has been observed in various neural networks across multiple spatiotemporal scales from locally occurring Hebbian plasticity to global synaptic scaling or homeostatic intrinsic plasticity (Turrigiano et al., 1998; Turrigiano and Nelson, 2004; Naudé et al., 2013; Toyoizumi et al., 2014). The impact of locally occurring homeostatic plasticity (Naudé et al., 2013) and how global homeostatic plasticity regulates neural network dynamics (Zierenberg et al., 2018) has been simulated in biological recurrent networks. However, it has not been tested in ANN to improve the performance, and no attempt has been made to find a similar concept in the current ANN optimization algorithm. Recent experimental confirmation also supports the idea that a neural network utilizes a plasticity rule that maximizes information (Toyoizumi et al., 2005) or minimizes free energy (Isomura and Friston, 2018; Gottwald and Braun, 2020; Isomura et al., 2022). Additionally, considering that the wiring between neurons requires metabolic resources in the BNN, as mentioned in the NAS and structural learning sections, we can also define the cost function that includes the constraints introduced by limited physical resources (Chen et al., 2006; Tomasi et al., 2013; Rubinov et al., 2015; Goulas et al., 2019). Although the target functions for a neural network to optimize are explicit in these examples, how the optimization results in learning a cognitive task remain elusive. However, they have inspired the ANN method to approach the learning of relationships among data to approximate the probability distribution of inputs or latent variables, which is an example of an unsupervised learning paradigm (Goodfellow et al., 2016; Pitkow and Angelaki, 2017). On the other hand, supervised learning can be defined more easily by quantifying the difference between the function to learn and the current state of a neural network, which is generally called the loss function in an ANN (Goodfellow et al., 2016). The strategy for minimizing the loss function and assigning the adjustment of each weight is characterized by a backpropagation algorithm (Rumelhart et al., 1986). While no explicit evidence has been found that the brain uses error backpropagation for learning, a hypothetical learning algorithm class, “neural gradient representation by activity differences (NGRAD),” has been suggested, which states that the information of activity difference is reflected as synaptic change, driving the learning or behavioral change of the network (Lillicrap et al., 2020). Considering that the backpropagation algorithm in ANN and error-dependent learning are not directly comparable because of the difference in encoding (scalar value vs. spikes) and the questionable existence of mandatory symmetric backward connections in BNN, organized feedback of error or target information is necessary for the implementation of NGRAD in a biologically plausible neural network (Guerguiev et al., 2017; Sacramento et al., 2018; Whittington and Bogacz, 2019; Lillicrap et al., 2020; Fernández et al., 2021). In a large neural network with physical constraints, relying only on the global feedback information provided through the environment is inefficient because of the long delay (Nijhawan, 2008; Foerde and Shohamy, 2011; Cameron et al., 2014). For example, when an animal tries to visually follow a fast-moving prey, moving the eyeballs at the proper speed and forming a proper percept without mental preparation by predicting sensory consequences is difficult (Greve, 2015; Palmer et al., 2015; Sederberg et al., 2018). Therefore, a neural system is known to utilize predictive coding, and the prediction error may be an appropriate teaching signal for optimizing each component in a hierarchical neural network (Rao and Ballard, 1999; Millidge et al., 2022; Pezzulo et al., 2022). A recent study theoretically suggested and experimentally validated that even a single neuron can predict future activity and use a predictive learning rule to minimize surprises; this is derived from a contrastive Hebbian learning rule (Luczak et al., 2022). Thus, this study has important implications for the bottom-up principle of local learning rules to form a learning algorithm for intelligent agents. The neuromodulatory system can participate in slower feedback or more implicit teaching signals (Johansen et al., 2014; Liu Y. H. et al., 2021; Mei et al., 2022). In fact, the three-factor rule constructed by simply adding a factor, such as neuromodulation, to pairwise synaptic plasticity can include diverse information about reward or learning hyperparameters (Gil et al., 1997; Nadim and Bucher, 2014; Łukasz Kuśmierz et al., 2017; Brzosko et al., 2019). Given the experimentally examined role of neurotransmitters in the neuromodulatory system and the local physiological dynamics affected by such neurotransmitters, the brain's mechanism of dealing with vast amounts of information from the natural environment can be explained by a combination of diverse modulatory inputs and the distinctive distribution of receptor subtypes (Noudoost and Moore, 2011; Rogers, 2011; Fischer and Ullsperger, 2017; Doya et al., 2021; Cools and Arnsten, 2022). Investigating global optimization algorithm and understanding it across multiple scales is important not only for neuroscience pursuing the answer of mechanisms of neural processes in the brain but also for constructing a better biologically plausible neural network capable of “general intelligence.”

## 3. Outcome of optimization: single computational unit properties

As Cajal's (1888) confirmation of the neuron doctrine implied, McCulloch and Pitts's (1943) theory of artificial neurons shaped the idea that a neuron is the single unit of computation, and a synapse is the single communication channel between two neurons. Although neurons and synapses have been intensively studied, several fundamental questions remain to be answered, including those regarding the computational roles of neuronal and synaptic properties. In ANN, a representative precedent, such as introducing a rectified linear unit (ReLU; Fukushima, 1975; Nair and Hinton, 2010), helped dramatically advance the field. Because single computational units in BNN are largely unexplored owing to their diversity and nonlinear properties, carefully searching computationally influential properties may enable us to build better neural networks.

### 3.1. Representation of the activity and coding scheme of a single neuron

The governing dynamics of the electrical properties of a neuron have been well-described and integrated into Hodgkin and Huxley's (1952) monumental work. This set of nonlinear differential equations can regenerate the dynamic excitability and action potential firing. A simpler description of the dynamics using the leaky integrate-and-fire model (Hill, 1936) can be utilized to reduce the complexity and extend the applicability to various types of firing patterns. In addition, direct reverse engineering of the spike parameters was successfully implemented (Izhikevich, 2003). In these neuronal models of the BNN, two distinctive aspects were noticeable, when compared with the ANN. One is that a set of continuous-time differential equations describes neuronal activities, and the other is that there is no explicit activation function except in the integrate-and-fire model and its variants. Although the information encoded in the spiking dynamics along continuous time in the BNN is not yet fully understood, several strategies that the BNN may utilize have been investigated. The well-known dichotomy of such strategies is the rate vs. temporal code (Gerstner et al., 1997; Guo et al., 2021). The rate code encodes target information using the firing rate, corresponding to a neuron's positive scalar value encoding in ANN. Temporal coding refers to an encoding strategy that utilizes the timing of spikes, and the specific coding scheme can vary depending on the time a neuron uses to represent information. For example, a period of silence is a candidate for inter-spike interval coding or time-to-first-spike coding (Dayan and Abbott, 2001; Park et al., 2020; Guo et al., 2021), or the absolute timing of multiple sparse spikes can be used to convey information under a proper decoding scheme (Comşa et al., 2021). The other aspect of the coding strategy, which extends the capacity for encoding, is to deploy a population of neurons to represent the information (Averbeck et al., 2006; Panzeri et al., 2015; Pan et al., 2019). Because the spiking patterns in a population of neurons can be statistically interpreted by considering each spike in each neuron as a sample of a specific random variable, an abundant representation form can be implemented. Different types of information can be conveyed through multiplexing by alternating coding schemes or mixing up heterogeneous neurons in a population (Harvey et al., 2013; Akam and Kullmann, 2014; Lankarany et al., 2019; Jun et al., 2022). For example, a sensor that waits for sparsely occurring inputs of various intensities can encode the input by timely bursting spikes upon an input arrival (Guo et al., 2021). Such a strategy is advantageous for richer dynamics and encoding capacity as well as lower power consumption by considering silence (off period) as another piece of information (Cao et al., 2015; Pfeiffer and Pfeil, 2018). Therefore, spiking neural networks (SNN) has become an essential type of ANN and are widely utilized in neuromorphic engineering (Kornijcuk et al., 2019; Kabilan and Muthukumaran, 2021; Parker et al., 2022). Because various models can describe a neuron's spike activity and each spike can represent distinctive information depending on the coding scheme, we can expect a much larger diversity of neuronal activation processes compared to ANN. Exploring various coding schemes with diverse temporal and populational spike patterns (Comşa et al., 2021; Guo et al., 2021) and heterogeneous distribution of diverse types of neurons (Stöckl et al., 2022) is necessary to represent complex information better and build more biologically plausible neural networks. Diverse types of neurons and their computational impacts have been tested and have demonstrated better performance in typical ANN by varying the type of activation function (Lee et al., 2018). Although groundbreaking improvements are rarely achieved by changing the activation functions in the deep learning field (Goodfellow et al., 2016), combinations of representations of activities in a neuron (spike), consequential spike-based synaptic plasticity (spike-timing-dependent-plasticity and spike-driven synaptic plasticity), various coding schemes (temporal, rate, population, and phase), and heterogeneous neuronal types have not yet been fully examined.

### 3.2. Dale's principle and input balance

Although the strongest interpretation of Dale's principle, which indicates one neurotransmitter type for one neuron, has become outdated and proven incorrect through accumulated experimental results (Osborne, 1979), it still offers an important framework for analyzing neural networks: the distinction between excitatory and inhibitory neurons (Eccles et al., 1976; Cornford et al., 2021). If we compare the synaptic efficacy in the BNN with that in the ANN, a direct correspondence can be found in the weights of the connection from one neuron to another. In contrast, the weight value in the ANN can vary between positive and negative values, and an input(presynaptic) neuron can include outward connections with both positive and negative weights unlike BNN neurons. Introducing the implications of Dale's principle to an ANN involves fixing a given neuronal identity to either an excitatory or inhibitory neuron, with the weights of its outward connections having the same signs. This is quite a strong constraint, but careful modification did not harm the network performance (Cornford et al., 2021) and provided more diverse computation (Tripp and Eliasmith, 2016) although there was no dramatic improvement in performance. Practical computational implications of the segregation of excitation and inhibition have not yet been established; however, by mathematical treatment of such a neural network, optimal dynamics of the neural network (Catsigeras, 2013) and efficient learning (Haber and Schneidman, 2022) have been carefully suggested as benefits. In BNN, it has long been suggested that a stable but sensitive representation of information can be achieved by balancing excitatory and inhibitory inputs, the so-called E-I balance (Denève and Machens, 2016; Hennequin et al., 2017). The implications of the E-I balance can be roughly explained by comparing it with other extremities. In an excitatory-dominant regime, excessive firing interferes with the expressibility of information by a neuron, whereas in an inhibitory-dominant regime, the frequency of firing drops, and the neuron cannot express the information that lies within a certain time scale. However, tightly balanced inputs can modulate a neuron to fire during a period of tiny temporal discrepancies between excitation and inhibition. Consequently, with an optimal number of firings, a neuron can efficiently represent multiple timescale inputs. The E-I balance has been restated and utilized to explain the performance and efficiency of biological neural circuit models (Denève et al., 2017; Zhou and Yu, 2018; Bhatia et al., 2019; Sadeh and Clopath, 2021) and the malfunctions of an imbalance regime (Sohal and Rubenstein, 2019). In ANN applications (Song et al., 2016; Ingrosso and Abbott, 2019; Tian et al., 2020), balanced inputs are utilized to optimize neural networks for better performance, with the advantages shown in BNN models. Because the concept of E-I balance covers a wide range of extents of balance (Hennequin et al., 2017), defining an alternative type of balanced network (Khajeh et al., 2022) is also possible. Considering that balancing is not just an artificial constraint but also the outcome of optimization (Trapp et al., 2018), applying excitatory-inhibitory segregation and its balance seem to be another prominent way to build better biologically plausible neural networks.

### 3.3. Morphological effect: dendritic computation

The types of neurons in a BNN are extremely diverse; one criterion is their heterogeneous morphology (Kepecs and Fishell, 2014; Cembrowski and Spruston, 2019). Unlike in a point neuron model, spatially separated input, processor, and output units are implemented as dendrites, somas, and axons, respectively, in a BNN. Thus, the morphological effect refers to the emerging directionality of information flow and the information contents affected by each unit. Notably, the input part (dendrite) is spatially distributed over a larger space than the output pathway (axon) that is often found as a minimally branched fiber consisting of somewhat homogeneous segments with small cross-sectional areas (Chklovskii, 2004). Hence, axonal fibers are expected to be primarily employed to faithfully convey the generated electrical signal (action potential) to distal postsynaptic neurons (Scott, 1975). In contrast, dendrites have many branches with thicker shafts capable of accommodating complex cellular organelles, except the nucleus. The complex branching pattern and spacious cytosol indicate that intracellular processes also occur in dendrites and may be spatially heterogeneous (Shemer et al., 2008; Dittmer et al., 2019). Because synapses are distributed across such heterogeneous substrates, information processed through synapses can be highly heterogeneous even when exposed to uniform presynaptic activity. Specifically, given that the change in shaft thickness varies with the branching or distance from the soma (Harris and Spacek, 2016), differentiating the electrical processing of each input from another is expected to depend on the location of the input (Guerguiev et al., 2017; Sezener et al., 2022; Pagkalos et al., 2023). A simple but remarkable aspect of such a structure and implication is the sequential processing of inputs from the distal location toward the soma, as the directionality of the information flow in a passive cable indicates. As a single action potential from a presynaptic neuron can be interpreted as a Boolean activation input, a recent study attempted to simplify the dendritic processing of many inputs as a layered neural network by adding active dendritic computation to the directionality (Beniaguev et al., 2021). This study highlighted the role of NMDA receptors capable of tuning the plasticity in each excitatory synapse and generating dendritic calcium spikes, which can be interpreted as the integration and firing of local inputs converging to a dendritic segment. Thus, each dendritic segment that generates spikes can be assumed to be a computing layer of converging Boolean inputs through a dendritic arbor, simplifying the complex information processing of a neuron and corresponding to the ANN. In neuroscience, there have been many observations of the active computation of dendrites via spike generation (Cook and Johnston, 1997; Poirazi and Mel, 2001; London and Häusser, 2005; Johnston and Narayanan, 2008). These examples also imply that various types of inputs are spatially and functionally segregated on distinctive branches or dendritic segments (Wybo et al., 2019; Francioni and Harnett, 2022); therefore, a neuron can work as a functional unit capable of more diverse performance than a point neuron. Because of the additional nonlinearity compared to a model point neuron, better expressibility can be expected (Wu et al., 2018), and electrical compartmentalization and active dendritic properties can be applied to ANNs (Chavlis and Poirazi, 2021; Iyer et al., 2022; Sezener et al., 2022). The segregated electrical properties also indicate that homeostatic control can occur separately in distinct dendritic branches (Tripodi et al., 2008; Bird et al., 2021; Shen et al., 2021). Such an adjustment of weights in each dendritic branch toward a certain homeostatic level is similar to the normalization step in ANN (Shen et al., 2021), which also improves learning in sparsely connected neural networks, such as BNN (Bird et al., 2021). The typical structure of a cortical pyramidal neuron consists of two distinctive directions of dendritic outgrowth from the soma: basal and apical dendrites (DeFelipe and Farias, 1992). These differ from each other not only in the direction of growth but also in the branching pattern. Additionally, owing to the vertical alignment of the dendrites of a cortical pyramidal neuron across the cortical laminar layer structure, basal and apical dendrites are exposed to inputs at different layers (Park et al., 2019; Pagkalos et al., 2023). Different branching patterns indicate distinctive information processing in the dendrites, as shown in the aforementioned study. Different input contents combined with different processing methods imply that diverse computations can occur at the microcircuit level, comprising several neurons. One remarkable application of this property is the assumption that a neuron processes both feedforward and feedback inputs, simultaneously. By postulating that error-conveying feedback and feedforward inputs containing external information are separately processed in distinct dendritic branches, the problem of credit assignment can also be explained (Guerguiev et al., 2017; Sacramento et al., 2018), as discussed in Section 2.2.3. Considering that in the biophysical model of a neuron, spontaneous orchestration of the dendritic properties of a neuron to learn a nonlinear function has been identified (Bicknell and Häusser, 2021), the computational implication of dendritic computation is no longer an assumption from the observation of morphology but becomes an essential governing principle of a single neuronal information processing.

## 4. Outcome of optimization: network architecture

Because single biological computing units exhibit numerous unexplored properties, large-scale combinations of these properties may enable neural networks to reveal complexities that can significantly affect neural network functions (Hermundstad et al., 2011; Braganza and Beck, 2018; Navlakha et al., 2018). The complexity that underlies the BNN emerges from other characteristics, such as high heterogeneity (Liu, 2020), overall sparse connectivity (Eavani et al., 2015; Cayco-Gajic et al., 2017), and hierarchical modularization (Meunier et al., 2010; Hilgetag and Goulas, 2020; D'Souza et al., 2022).

### 4.1. General distinctive characteristics of the network structure in BNN

The construction and maintenance of hard wiring from one neuron to another involve metabolic and volumetric costs (Chen et al., 2006; Tomasi et al., 2013; Rubinov et al., 2015; Goulas et al., 2019); thus, in a BNN, it is difficult to imagine dense connections, as in an ANN, where we often encounter fully connected layers. The sparse connectivity in the BNN inspired the construction of a lightweight deep learning architecture (Wang C. H. et al., 2022). Model compression by the sparsification of connectivity has led to a large reduction in power consumption, while minimizing performance reduction (Han et al., 2015; Barlaud and Guyard, 2021; Hoefler et al., 2022) and improving performance (Luo, 2020). Identifying the sweet spot between optimized sparsity and performance is the next challenge (Hoefler et al., 2022), and as explored in Section 2, EA may be a suitable choice (Mocanu et al., 2018). As the outcome of a properly chosen sparsification algorithm, the connectivity map of an optimal sparse network also directly improves neural network interpretability because the putative essential connections to process the task are presumably spared, while the unnecessary connections are pruned (Hoefler et al., 2022).

Combining high heterogeneity with sparse connectivity results in modular structures (Mukherjee and Hill, 2011; Miscouridou et al., 2018), and the highly modular structure of the BNN shows the same set of advantages as sparse connectivity. The modular structure can be interpreted as an aggregation of computational units employed for the same function. These units (neurons) are usually located near each other and activated at the same developmental stage, which implies that the general wiring principle in BNN, involving activity- and distance-dependent wiring, may shape the modular structure (van Ooyen, 2011). Contrary to the constructive algorithm by the developmental process, learning-based decomposition into modules is also possible (Kirsch et al., 2018; Pan and Rajan, 2020), enhancing the interpretability and convenience of troubleshooting. In addition, connecting modules that perform distinct functions enables the task-specific design of a comprehensive neural network (Amer and Maul, 2019; Michaels et al., 2020; Duan et al., 2022). Because each module can be considered a building block of a neural network, the evolutionary strategy may perform best in identifying the entire architecture optimized for a certain task (Clune et al., 2013; Lin et al., 2021). Such a strategy eventually maximizes the functional performance of each building block and implies scalability without interfering with the performance of other modules (Ellefsen et al., 2015), while maintaining a minimal number of additional connections. This example is directly related to the answer regarding how the brain can acquire and store multiple memories by not harming old ones and not interfering with new learning with a finite number of hardware units. Such a problem can be characterized by catastrophic forgetting and interference during continual learning, and many candidate mechanisms that the brain may utilize to solve these problems have been suggested (Hadsell et al., 2020; Jedlicka et al., 2022). The modular structures combined with the sparse representation are a more intuitive solution than others because it assigns each piece of information to a separate hardware, implying faster and more precise access to the memory unit. Although the number of neurons and synapses is still not enough to afford all the information which an intelligent agent learns during their lifespan, the modular structure may play a key role in efficient continual learning by harnessing other mechanisms regarding common information.

### 4.2. Connectivity in a specific brain region

Considering that the largest scale of the module structure is the functional modularization of the brain into each brain region, the most straightforward way for AI to acquire a certain function is to copy the connectivity of the specific brain region that regulates that particular function. Although the current brain-wide or regional wiring map is far from completion, several brain areas are known to have relatively organized connectivity and regulate well-defined functions.

One of these brain regions is the cerebellum. Because of its relatively simple and organized structure, the cerebellum was the first target for computational modeling as attempted by Marr (1969); Albus (1971). Major streams of the cerebellar information processing can be divided into a feedforward network through granule cells and Purkinje cells, and a feedback connection from inferior olive where a part of the cerebellar outputs projects. Because the feedforward stream conveys the information from the cortex and the olivary feedback sends the error between sensory feedback and sensory prediction, the Purkinje cell where these streams converge has been assumed to adapt to minimize the error signal (Raymond and Medina, 2018). This conjecture based on the structure was directly applied to a cerebellar model articulation controller (CMAC; Albus, 1975), which is based on the fact that the cerebellum is involved in smooth motor control. CMAC is still utilized with modifications (Tsa et al., 2018; Le et al., 2020). Because the cerebellum is not a sole motor controller, the whole motor control process should be analyzed by including the initial command generator and motor plant. Considering that the cerebellum receives inputs from the cerebral cortex through pontine nuclei and propagate outputs to the cortex through deep cerebellar nuclei to thalamic projection, the loop between the cortex and the cerebellum can be interpreted as the continuous corrector of the ongoing motor control. The importance of such a brain-wide loop structure in which a cerebellum is involved has been recently raised and integrated into ANN models (Iwadate et al., 2014; Tanaka et al., 2020; Boven et al., 2023). Furthermore, in recent decades, our understanding of the cerebellum and its functions has deepened considerably, including the non-motor output from the cerebellum (Kang et al., 2021; Hwang et al., 2023) and multi-dimensional structural organization (Apps et al., 2018; Beckinghausen and Sillitoe, 2019). Although, currently, we barely understand the detailed network architecture underlying such diverse functions and gross anatomy, further research will lead us to implement the control of broad behavioral modality through the cerebellum.

The hippocampus is also a brain area that deserves a brief introduction here. The hippocampus has well-defined functional roles in episodic memory and spatial cognition, and the overall information flow across the sub-regions is also known (Bird and Burgess, 2008; Kovács, 2020; Li et al., 2020). The improved artificial memory system has drawn more attention regarding the memory mechanisms and implementation of the memory circuit (Berger et al., 2012; van de Ven et al., 2020). Traditionally, the auto-associative connectivity in CA3 was characterized and inspired the Hopfield-type memory network (Hopfield, 1982; Ishizuka et al., 1990; Bennett et al., 1994). In addition, considering that the well-known connections from CA3 to CA1 roughly form a hetero-associative network, the stored information can migrate along the feedforward organization within the hippocampus (Graham et al., 2010; Miyata et al., 2013). However, because such associative memory structures are known to have limited capacity (McEliece et al., 1988; Kuo and Zhang, 1994; Bosch and Kurfess, 1998), additional structure or functional extension is necessary to reach the biological memory capacity level which can store dense information during a whole lifespan. Considering that the hippocampus receives inputs from the cortex through the dentate gyrus and projects back to the cortex through CA1 output, the interaction between the hippocampus and the cortex has been suggested to have the role of the memory buffer and consolidation (Rothschild et al., 2017). In addition to the modular structure with sparse representation as mentioned in a previous section, working mechanisms of this interplay have been suggested, such as generative replay and metaplasticity (Hadsell et al., 2020; van de Ven et al., 2020; Jedlicka et al., 2022), by resolving how to efficiently reorganize the representation of the information with time across the network. Considering that these mechanisms are inferred by the observations of both functional data and the architecture, the applications of these mechanisms to ANN (Hadsell et al., 2020; van de Ven et al., 2020; Wang L. et al., 2022) propose more intense collaboration between neuroscience and AI engineering toward a neural network design containing both bioplausibility and better performance.

Besides the cerebellum and hippocampus, other unexplored brain areas can be used to build biologically plausible neural networks. Since the recent advances in neuroscience have revealed not only the map of structural and functional connections within a region and across regions but also the relationship between the structure and function, careful imitation of other brain areas with proper simplification and interfacing will be demanding.

## 5. Discussion

### 5.1. The goal and limitation of a bottom-up approach

Putting aside the hardware issue and the question of intrinsic infeasibility, whether copying a BNN by artifacts can generate the intelligence possessed by a human or animal directly requires the goal and limitation of a bottom-up approach. While we have partially reviewed recent advances in bottom-up approaches to construct neural networks, it should be noted that replacing only a certain part of the ANN with one from the BNN usually does not improve the performance measured by the criteria for ANN. In other words, if we introduce a new concept from a BNN, the entire framework must be changed. For example, to utilize spike-timing-dependent plasticity, a change from an ANN to an SNN is necessary, and consequently, the task design needs to be modified. For certain tasks, such as predicting the digit annotation from the images drawn from the MNIST dataset (LeCun et al., 2010) after supervised learning, the ANN can achieve the best precision, while the SNN may not be able to outperform it. However, when implemented in hardware, SNNs have a considerably greater advantage in terms of power consumption, as observed in modern neuromorphic hardware (Cao et al., 2015; Pfeiffer and Pfeil, 2018; Cui et al., 2019; Kornijcuk et al., 2019; Kabilan and Muthukumaran, 2021; Parker et al., 2022). In addition, as mentioned in Section 3.1, SNNs may have the advantage of dealing with intermittently activated inputs (Pfeiffer and Pfeil, 2018). Thus, this example prompts us to build an alternative interpretation that the advantages of a certain neural network can vary with the type of problem that the neural network must solve.

**Figure 2:**
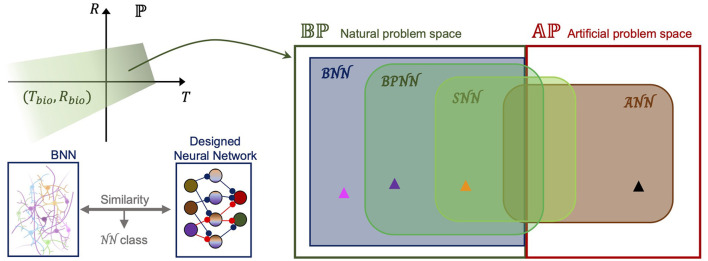
Problem spaces and cover sets by neural network designs. **(Left top)** In the entire problem space (ℙ), natural problems can be defined as the green region where both the task (*T* which includes the dataset and the goal) and the performance measure (*R* which includes the efficiency measure, the number of required computations, and platforms to perform the task) are within the biological range. **(Left bottom)** Neural network class can be defined by comparing a designed neural network with a biological neural network. The similarity decides its class. **(Right)** Binary division of the problem space into the natural problem space (*Bℙ*) and artificial problem space (*Aℙ*) as aforementioned. Neural network classes are: ANN, artificial neural network; BNN, biological neural network; BPNN, biologically plausible neural network; SNN, spiking neural network. The black arrowhead represents the problems for ANN supremacy. Magenta: BNN supremacy; Purple: BPNN supremacy; Yellow: SNN supremacy, compared with ANN.

To generalize these observations, first, we defined “the problem space,” which is the set of problems that neural networks try to solve. “A problem” (*P*) is defined by the task itself (*T*), including the dataset and goal, and by the performance measure of the task (*R*), including the efficiency measure like power consumption or the number of required computations or platforms to perform the task. By mapping *P*, these attributes represent a point in the problem space ℙ. For a certain problem, if we set the naturalistic task and try to achieve the evaluation measure in the range of humans or animals, the problem is a point in the “natural problem space” in Figure 2. By simply assuming that there is a subset of ℙ that consists of points mapped from the biological range of *T, R* (*T*_*bio*_, *R*_*bio*_), the set of natural problems (natural problem space) can be defined as Bℙ as follows:


(1) $$B\mathbb{P}=\{y\in \mathbb{P}|y=P(T_{bio},R_{bio})\},$$

and all the non-natural problems belong to the “artificial problem space” (*Aℙ*). For example, tracking fast-moving prey without intensive pretraining is a natural problem, but identifying a fingerprint from a vast database is an artificial problem. In fact, determining a problem type can be taken care of by neuroscience, specifically, by a top-down approach, because it ultimately determines whether this task is one of what can be done with the brain, in Turing's (1948) terms.

These problems in *Bℙ* or *Aℙ* can be solved using neural networks; however, the coverage differs depending on the class of a neural network. ANNs have shown powerful performance, at least for problems in *Aℙ*, and have also been employed to solve natural problems by reducing power consumption and minimizing training. Thus, as shown in the Venn diagram in Figure 2, the ANN class covers some natural problems and a larger part of the artificial problem space. On the other hand, the neural network class SNN has been utilized to solve more natural problems than artificial problems. For instance, by hardware implementation, an SNN can greatly reduce the required resources with similar precision to an ANN in image classification, but an ANN can show better optimization for performance on a typical computer after training with a large dataset. Therefore, as shown in Figure 1, the SNN and ANN intersect in both problem spaces, and the intersection in the natural problem space is a subset of the SNN. By contrast, the BNN class is a subset of the natural problem space that covers most of the *Bℙ* region. Because we defined natural problems as those that the brain can solve, it is reasonable to assume that a BNN as a unit of the brain can be employed to process most natural problems not covered by other classes of neural networks. We would like to call the relative complement in BNN the “BNN supremacy regime,” which is the actively used phrase in quantum computing (Arute et al., 2019). Thus, when building a biologically plausible neural network, the task, its performance measure, and the neural network architecture need to be changed to prove a better performance of a designed neural network than an ANN. Given the assumption that the class of biologically plausible neural networks, BPNN, is defined by the similarity to BNN architecture, our practical short-term goal is not only to construct a BNN-like architecture but also to demonstrate the “BPNN supremacy” by finding a proper problem in *Bℙ*. There have been attempts at formalization with similar motivations on SNN (Maass, 1996; Kwisthout and Donselaar, 2020) or ANN (Balcazar et al., 1997), and solving the shortest path problem is a problem in the relative complement of ANN in SNN that has been discovered (Aimone et al., 2021). Eventually, formalization and a mathematical approach are necessary to better define the problem spaces and investigate the spectrum in a set.

### 5.2. The role of neuroscience in the bottom-up approach to explore the BNN supremacy regime

**Figure 3:**
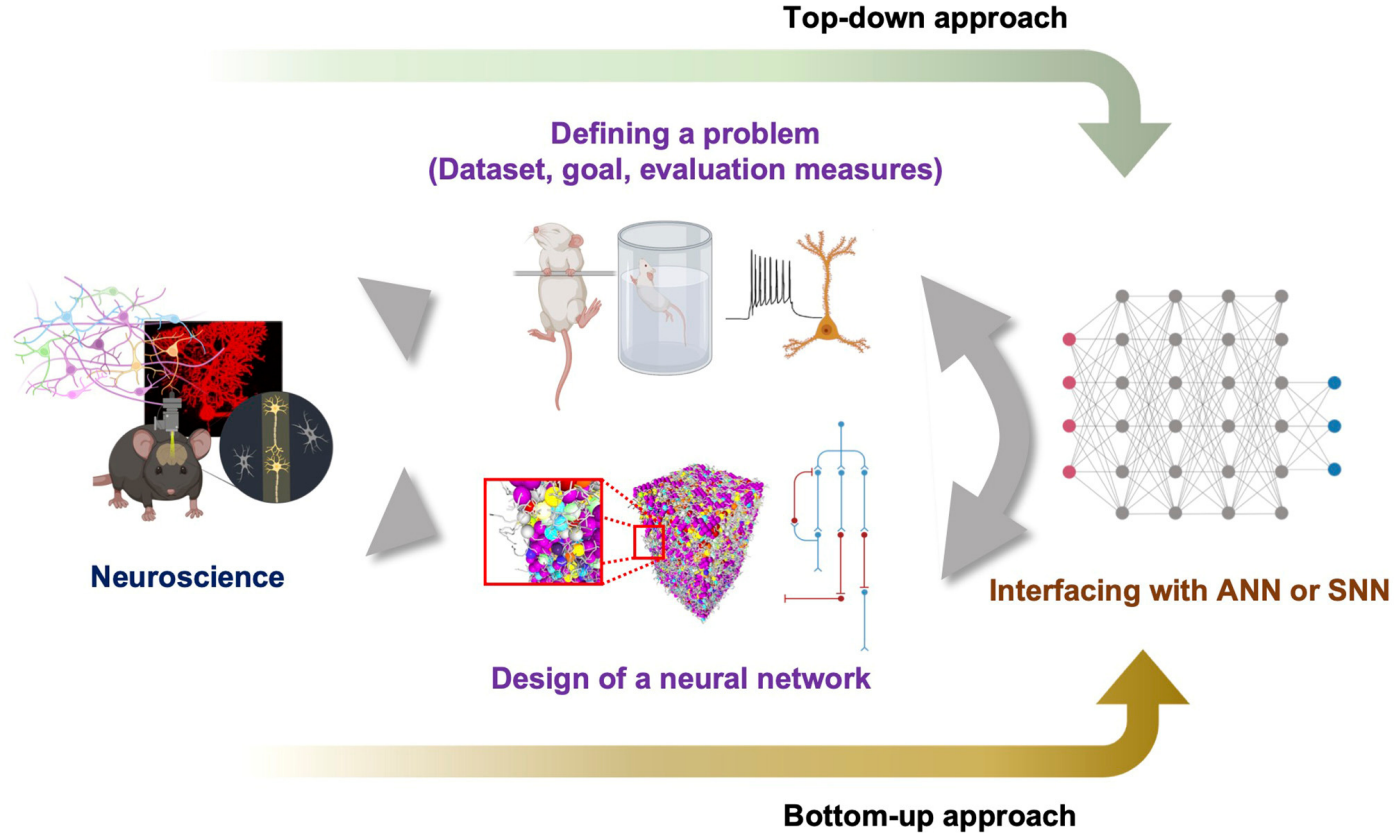
Suggested pipeline to explore problem spaces and proper design of neural networks. The top-down approach defines the problem to solve based on the findings of neuroscience and the bottom-up approach designs a neural network. To determine whether the problem can be solved by a designed neural network without slow search, both need to be hybridized with feasible neural networks such as ANN or SNN.

How can we discover points in the problem spaces, specifically within the BNN or BPNN supremacy regime? Does a proper design of a BNN or BPNN always exist for certain problems? We do not have a concrete formalization scheme or rough map of problem spaces to answer these questions fundamentally using mathematical proofs. Furthermore, we do not have any information regarding the proper design of neural networks. Thus, we suggest the pipeline shown in Figure 3, which starts with neuroscientific discoveries and shows how to define a problem specifically in the natural problem space. Accumulated data related to neuroscience can help define the task goal and the corresponding dataset to train neural networks through a top-down approach that specifically pursues the neural network mechanism by starting from observations at the level of the cognitive behavior of an intelligent agent. Thus, a top-down approach may be able to define a point in the problem space and distinguish between points in the *Bℙ* and *Aℙ*. Simultaneously, a bottom-up approach may enable the design of a neural network by combining many essential properties of the targeted BNN with the generalized principles of a neural network. However, because the definition of the problem in *Bℙ* may be too complicated to completely formulate, and it is difficult to judge whether the designed neural network can solve the problem before emulation, the defined problem and the hypothesized neural network design should be embedded in an already built scheme such as ANN or SNN to utilize feasible engineering techniques. Such hybridization is necessary for estimating the solvability of the problem without a full emulation. We speculate that persistent exploration by following the suggested pipeline will fill the information in the diagram shown in Figure 2, which can eventually enable a formal investigation to derive the set boundary. We believe that this type of slow but straightforward bottom-up approach and collaboration with a top-down approach and interfacing with current ANN will help us to light up the way to build a thinking machine like the human on the concrete foundation of neural circuit principles. Moreover, this pipeline could promote improved communication between neuroscience and AI engineering.

## Author contributions

IJ and TK searched and analyzed the references. IJ wrote the draft. TK arranged the original idea and revised the draft. All authors contributed to the article and approved the submitted version.
